# Spine surgery cost reduction at a specialized treatment center

**DOI:** 10.1590/S1679-45082013000100018

**Published:** 2013

**Authors:** Dan Carai Maia Viola, Mario Lenza, Suze Luize Ferraz de Almeida, Oscar Fernando Pavão dos Santos, Miguel Cendoroglo, Claudio Luiz Lottenberg, Mario Ferretti

**Affiliations:** 1 Hospital Israelita Albert Einstein, São Paulo, SP, Brazil

**Keywords:** Costs and cost analysis, Cost-efficiency analysis, Spine/surgery, Orthopedic procedures

## Abstract

**Objective:**

To compare the estimated cost of treatment of spinal disorders to those of this treatment in a specialized center.

**Methods:**

An evaluation of average treatment costs of 399 patients referred by a Health Insurance Company for evaluation and treatment at the Spine Treatment Reference Center of Hospital Israelita Albert Einstein. All patients presented with an indication for surgical treatment before being referred for assessment. Of the total number of patients referred, only 54 underwent surgical treatment and 112 received a conservative treatment with motor physical therapy and acupuncture. The costs of both treatments were calculated based on a previously agreed table of values for reimbursement for each phase of treatment.

**Results:**

Patients treated non-surgically had an average treatment cost of US$ 1,650.00, while patients treated surgically had an average cost of US$ 18,520.00. The total estimated cost of the cohort of patients treated was US$ 1,184,810.00, which represents a 158.5% decrease relative to the total cost projected for these same patients if the initial type of treatment indicated were performed.

**Conclusion:**

Treatment carried out within a center specialized in treating spine pathologies has global costs lower than those regularly observed.

## RESUMO

**Objetivo:**

Comparar o custo estimado do tratamento de patologias da coluna aos verificados para esse tratamento dentro de um centro especializado.

**Métodos:**

Foi realizada a avaliação dos custos médios de tratamento de 399 pacientes encaminhados por uma fonte pagadora para avaliação e tratamento no Centro de Referência de Tratamento da Coluna Vertebral do Hospital Israelita Albert Einstein. Todos os pacientes apresentavam indicação de tratamento cirúrgico antes de serem encaminhados para avaliação. Do total de pacientes encaminhados, apenas 54 foram submetidos a tratamento cirúrgico e 112 a tratamento conservador com fisioterapia motora e acupuntura. Os custos dos dois tratamentos foram calculados com base em uma tabela previamente acordada de valores de reembolso para cada etapa do tratamento.

**Resultados:**

Os pacientes tratados de forma não cirúrgica apresentaram custo médio de tratamento de R$ 3.245,16, enquanto os pacientes tratados com cirurgia apresentaram custo médio de R$ 36.590,16. O custo total estimado da coorte de pacientes tratados foi de R$ 2.339.326,09, o que representa uma diminuição de 158,5% em relação ao custo total projetado desses mesmos pacientes, caso fosse realizada a indicação inicial de tratamento.

**Conclusão:**

O tratamento realizado dentro de um centro especializado em tratamento de patologias da coluna apresenta, globalmente, custos menores do que os observados regularmente.

## INTRODUCTION

Diseases that affect the spinal column are common, and in most of them, conservative treatment (utilizing mechanical rehabilitation) produced good results. However, when conservative treatment fails, a significant number of patients may need surgical interventions.

Surgical treatment of the spine encompasses a large number of procedures, which use resources in diverse ways. The term “spine surgery” may represent a simple decompression of the medullar canal (as, for example, a micro discectomy for a herniated disc) or even arthrodesis (fusion) of various levels of the spine.

The advent of new technologies in equipment and implants makes innovations possible in carrying out surgical procedures. On the other hand, these innovations have increased the costs of surgeries, making it a point of concern for public and private healthcare services in the entire world.

Currently, there is no definition of cost-effectiveness that justifies the use of high-cost technologies for spinal surgeries^(1)^. In the United States, lumbar discectomy is the surgical procedure performed most frequently for patients with symptomatic herniated discs^(2)^. Assessment of cost-effectiveness of surgical treatment of herniated discs is very difficult, since it needs to take into consideration the population that is being evaluated and the social impact of the disease on the work force (indirect costs)^(3,4)^. Many governments, such as that of Holland, recommend diminishing indirect costs to lead to a substantial reduction in the cost of treating spinal disorders^(5)^.

The various types of conservative treatment by physical therapy also show different efficacy and costs. For chronic lumbago, there are no great differences between efficacy of usual physical therapy in the outpatient´s clinic from vertebral stabilization physical therapy and Pilates exercises – these have proven more cost-effective in the control of pain than traditional physical therapy ^(6)^. As to the use of intensive physical therapy or regular protocols, there is also no difference as to cost-effectiveness of treatments^(7)^.

Indication of arthrodesis surgery for degenerative disc pathologies has not yet presented irrefutable scientific evidence. Thus, this type of procedure is constantly questioned as to its cost-effectiveness in treating the disease^(8)^.

## OBJECTIVE

The objective of this study was to evaluate the estimated costs of treatment of spinal diseases, when they are performed within a center for specialized treatment in comparison with those usually performed in the healthcare market.

## METHODS

The data from 419 patients referred to the Reference Center for Spinal Treatment of Hospital Israelita Albert Einstein (HIAE), during the period from May 1^st^, 2011, to April 30^th^, 2012. All patients presented with indications for surgical treatment and had been referred, by the healthcare insurance companies, for a second medical opinion at HIAE. Patients were contacted by the Health Insurance companies requesting the evaluation of the second opinion. After this contact, the hospital was advised as to the status of the patient and made contact with him/her to schedule the medical visit.

The data utilized were not derived from medical records, but from administrative forms and those of the Program of Specialties of the Muscle-Skeletal System that manages the Reference Center for Spinal Treatment at the HIAE. In the preparation of this project, we included the amount of patients seen at each step of the process and the estimated cost of these patients.

### Treatment protocol

Protocols for evaluation and treatments proposed by the Program for Spinal Treatment at HIAE began with two medical visits, one with a physiatrist and the other with an orthopedic surgeon. Each physician made their evaluations independently, as well as their indication (conservative or operative treatment, with indication of the evaluation by a spinal surgeon of the HIAE team). Patients that presented with divergent indications from the orthopedic surgeon and physiatrist were referred for evaluation by the surgeon. The patients with indications for conservative treatment (two converging and independent opinions) were informed that, if they accepted the indication, they could undergo rehabilitation at the HIAE rehabilitation center. At no point in the evaluations were the patients obliged to accept the treatment proposed and/or to migrate to be treated at HIAE.

The HIAE rehabilitation protocol for spinal diseases was designed with 20 sessions of motor physical therapy (two periods of 10 sessions with an intermediate reevaluation clinical visit, focused on kinesiotherapy and physical analgesic means) and six sessions of acupuncture (analgesic).

From May 2011 until March 2012, care was individualized (one physical therapist for each patient in 50-minute sessions). This protocol persisted until March 2012, when after various evaluations at the institution, idleness was noted in the process. Since then, rehabilitation sessions have been done with one physical therapy professional and two concomitant patients. Patients who accept the treatment are also referred for acupuncture, with analgesic effect, in 50-minute sessions.

In the initial protocol, patients are reevaluated after the tenth rehabilitation session by the physiatrist. If symptoms improve, the patient is maintained in treatment for another 10 sessions. Patients whose symptoms do not improve are referred for evaluation by a spine surgeon from the HIAE team. Intermediary treatment assessment has been considered an issue of safety for the patient.

After the end of the 20^th^ physical therapy session, the patient might present without symptoms (cured, and is discharged), with partial improvement of symptoms (will continue at a rehabilitation center that is part of the healthcare plan network), or with no change (will be referred for evaluation by a spine surgeon of the HIAE team).

Patients with indication for surgical treatment are referred for assessment by one of the seven reference spine surgeons of HIAE. This group of doctors comprises specialists in Orthopedics or Neurosurgery, chosen from within the clinical staff of the hospital, based on their academic and clinical experience and their relation with the hospital. The choice of the surgeon to receive the case is made randomly.

### Follow-up protocols

All patients were submitted to functional evaluation by means of standardized questionnaires. The evaluations occurred during the first clinical visit, with the presence of the patient, and at the end of the first, third, and sixth months of treatment with telephone contacts.

### Costs

Costs of the treatments (values paid by the health insurance company) were estimated based on the sum of costs of each phase of treatment, which was based on previously agreed values between the hospital and the health insurance companies. Costs were calculated based on the complete evaluation protocol, but some patients did not go through all the evaluation processes, which may have caused an overestimate of the foreseen costs.

It was considered that information with the exact cost values individualized for each item of the hospital bill, both of the hospitals and the healthcare companies, was strategic and non-accessible data. For purposes of comparison, it was estimated that the mean costs in the pool of hospitals in Sao Paulo showed values similar to those negotiated between HIAE and the health insurance company, especially for this project.

For all patients, costs of treatment were considered as the initial clinical visits with the physiatrist and orthopedic surgeon. In patients treated conservatively, the complete set of 20 physical therapy and 6 acupuncture sessions were considered. Not all patients did the complete conservative treatment, since many improved during treatment and were discharged. For assessment of the total cost, even when overestimating the cost of conservative treatments, the entire treatment proposed was considered.

For patients treated surgically, the individualized values of each procedure were used. The arthrodesis procedure has a variation of costs, depending on the number of levels performed and of the topography (cervical or lumbar). For calculation purposes, the mean value charged for the arthrodesis was considered (Table 1).

**Table 1:** Composition of treatment costs estimated according to the type of treatment given (conservative and surgical). For each patient, his/her individual cost of treatment was estimated by the sum of items that make up their group

Conservative treatment	Surgical treatment
1 medical consultation with an orthopedic surgeon	1 medical consultation with an orthopedic surgeon
1 medical consultation with a physiatrist	1 medical consultation with a physiatrist
20 physical therapy sessions	1 medical consultation with a spine surgeon
6 acupuncture sessions	Value of the surgery performed: simple decompression OR arthrodesis (mean value) OR rhizotomy (mean value) OR infiltration

The data in reference to the initial surgical indication are not always precise, and many times are classified in a simple manner, such as arthrodesis and rhizotomy, with no reference to the quantity of levels. Hence a mean value for these procedures was observed, which was quantified in the same way for treatments in and out of HIAE.

### Sample description

Of the total 419 patients referred by the health insurance company, only 399 came for the first evaluation consultation. After the first assessment with the orthopedic surgeon and physiatrist, 218 patients, who presented with disorders with probable surgical etiology, were referred for care by a spine surgeon of the HIAE team and 181 patients were referred for conservative treatment.

Among the patients referred for conservative treatment, 96 did the treatment at the HIAE rehabilitation center, 16 did rehabilitation at another service different from HIAE, and 69 patients did not accept the treatment proposed or were excluded from the treatment program by the health insurance company.

As to the 218 patients referred for evaluation of a surgeon of the HIAE team, 56 did not accept having their evaluation at HIAE and 162 went through another medical consultation. After evaluation with the HIAE surgeon, 103 patients had indications for surgical treatment, while 41 patients were referred for conservative treatment (only 16 did it at HIAE); the other 18 did not accept the treatment proposed or were excluded from the program. Of the 103 patients with indications for surgical treatment, 54 were submitted to surgical procedure at HIAE and 49 underwent the treatment outside of HIAE (they returned to the physician who had previously treated them). Table 2 shows the segmentation of patients according to the type and location of treatment.

**Table 2:** Segmentation of the patients as per the type and location of the treatment

Treatment given	n (%)
Surgical treatment performed at HIAE	54 (12.9)
Surgical treatment performed at another service	49 (11.7)
Conservative treatment given at HIAE	112 (26.7)
Conservative treatment given at another service	41 (9.8)
Patients who did not accept the treatment proposed at HIAE	163 (38.9)
Total	419 (100)

HIAE: Hospital Israelita Albert Einstein.

Of the 419 patients with initial indications for surgery, only 103 were in fact treated surgically. The cost of treatment for these patients was structured as per the composition of table 1, considering the sum of the following values: cost of the (original) medical consultation with the orthopedic surgeon and physiatrist, cost of the initial evaluation of the spine surgeon and cost of the surgery performed. For the 153 patients treated conservatively, the cost of treatment was also structured based on table 1, representing the cost of the conservative treatment.

## RESULTS

**Figure 1:**
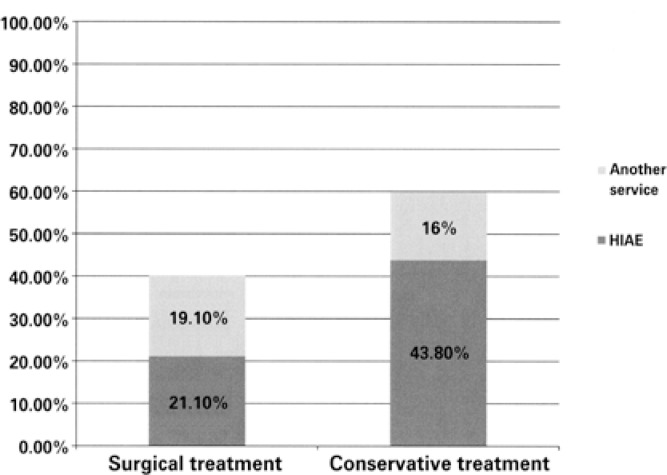
Patients who accepted the treatment proposed by Hospital Israelita Albert Einstein team (HIAE; n=256), classified by the type of treatment given and by the location where the treatment was given

Patients treated conservatively (n=153) represented a reduction of 36.5% in the volume of surgeries relative to the initial proposal (n=419). Figure 1 shows the patients who accepted the treatment proposed by the team of HIAE (n=256), divided by the type of treatment given and by the location where they received the treatment.

Patients treated surgically at HIAE (n=54) presented with a total estimated cost of US$ 1,002,826.00, with a mean cost per patient treated of US$ 18,576.00. The same cohort of patients, if the initial surgical indication were maintained, would represent a total cost of US$ 1,227,028.00, with a medium cost of US$ 22,723.00 per patient treated. In this group of patients, the cost reduction with the treatment given was 22.5%, in comparison with the initial treatment proposal.

Patients treated conservatively at HIAE (n=112) presented with a total estimated cost of US$ 184,305.00, with a mean cost per patient treated of US$ 1,650.00. The same cohort of patients, if the initial surgical indication were maintained, would have a total cost of US$ 1,840,977.00, with a mean cost of US$ 19,622.00 per patient treated. In this way, it was observed that the treatment proposed at HIAE brought an expense for the health insurance company 1.089% lower than the cost of the treatment proposed initially.

The total estimated cost of the treatment performed at HIAE was US$ 1,187,295.00, a value that differs from the initial estimated cost of this same cohort of patients by US$ 3,069,094.00, which represents a 158.5% reduction of the value that would be spent in case the treatment were performed in the manner proposed initially.

**Figure 2:**
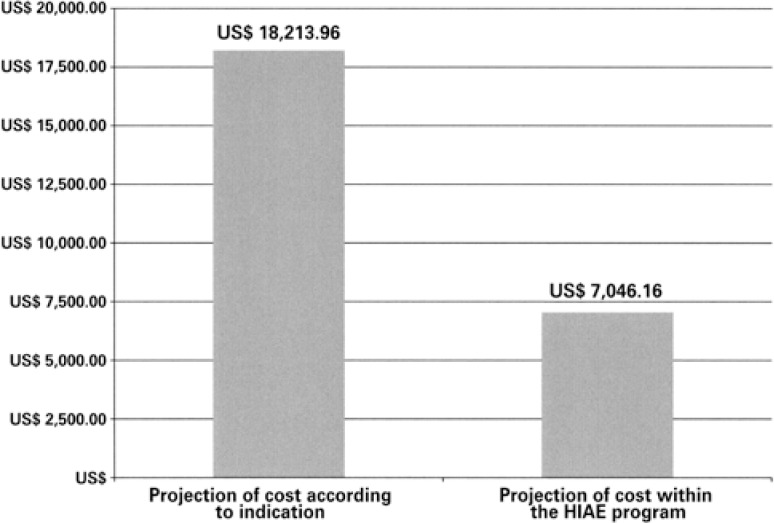
Projection of cost, per patient treated, as per the initial indication and according to the treatment proposed at Hospital Israelita Albert Einstein (HIAE), for the patients treated at this hospital (n=166)

Table 3 demonstrates the cost of the treatment given and of the treatment proposed initially for the patients treated at HIAE. Figure 2 shows the projection of cost per patient treated, as per the initial indication and according to the treatment proposed at HIAE, for the patients treated at HIAE (n=166).

**Table 3:** Cost of treatment given and of the treatment proposed initially for patients treated at Hospital Israelita Albert Einstein (HIAE)

Treatment given	n	Estimated cost of treatment given at HIAE	Cost of treatment initially proposed
Surgical treatment performed at HIAE	54	US$ 1,002,826.21	US$ 1,228,117.81
Conservative treatment performed at HIAE	112	US$ 184,304.45	US$ 1,840,976.49
Total	166	US$ 1,187,294.36	US$ 3,069,094.31

## DISCUSSION

The organization of a specialized center for spinal treatment contributes towards a reduction in indications of surgery. Within an organization structure, with well-defined flows and processes, the attending physicians have the security of knowing that if the conservative treatment fails, the patient is rescued and a new treatment may be quickly proposed. Additionally, the double initial evaluation (orthopedic surgeon, with a surgical profile, and physiatrist, with conservative profile) allow an early discussion as to the patient's status and a referral focused on the opinions of distinct professionals.

This type of structure is a contrast with the system traditionally used in Brazil, in which the physician sees the patient isolated in the office, refers him/her to a physical therapist (who often is various miles away), and due to a series of structural reasons, reevaluation only occurs days later.

The creation of centers specialized in spinal treatment should lead to specialization with greater efficiency. Kwon et al.^(9)^, however, have observed that in some specialized centers, there is an increase of costs, without necessarily an improvement in treatments. This occurs, primarily, due to the use of more sophisticated (and more expensive) diagnostic and treatment methods. This goes against the data of this study, since the use of a specialized center afforded a reduction in the number of surgeries and consequently, in the final cost of the treatment. This difference in results should be related to the fact that there was great variability in the treatment indicated by the center observed here relative to the initial indication of treatment.

The indications and costs of spinal surgery show a high rate of growth, without necessarily representing an improvement in results. Rihn et al.^(10)^ considered that the current standardization of treatments and the changein diagnostic methods and in measurements of evaluation of clinical follow-up may improve the results and the costs of treatments. The creation of the specialized center observed was based on these principles and early on revealed a drop in treatment costs.

This example can also be applied for other spinal pathologies, not only the degenerative. Patients with traumatic spinal cord lesions, when treated at specialized centers, show a much more favorable progress with lower morbidity and mortality, less time of hospitalization, and lower rates of complications relative to those treated by teams in non-specialized centers^(11,12)^.

For highly complex illnesses, treatment in specialized centers may afford better compliance of the patient with the rehabilitation protocols, allowing a closer control of all the patients and quicker interventions, in case the patient does not progress as expected. For the treatment of spinal disorders, HIAE chose to establish a specialized center, with a focus on the quality that the specialization of the entire team may add to the process.

Management of physical therapy treatment for acute lumbago or that related to activity by means of protocols based on classifications, instead of treatment based on conduct manuals and individual experience, proved less expensive and brought better clinical results^(13)^. This observation highlights the idea that centers with greater organization of the service have the conditions necessary to promote inexpensive treatments with better results for the patients ^(7)^.

Patients with herniated discs generally present with an indication for rehabilitation before the surgical treatment. Daffner et al.^(14)^ evaluated the costs of conservative treatment, from the diagnosis to therapeutic methods, in patients in whom this treatment failed, with the need for surgical intervention. The authors found mean costs of US$ 3,445.00 for the conservative treatment performed before the discectomy. Despite this value not being directly comparable to ours (due to different economic moments and different market situations), we agree with the authors that the conservative treatment should always be tried before the surgical. The relatively low costs of conservative treatment (when compared to surgical treatments) justify its use, since they may lead to an improvement of symptoms in many patients.

Surgeries for cervical stenosis, on the other hand, presented low cost-effectiveness in the two first postoperative years. Cervical arthrodesis surgery presented costs highly superior to those of simple decompression, without showing significant alterations in the Quality-Adjusted Life Year (QALY)^(15)^. The indication of arthrodesis should always be based on instability (primary or generated by decompression) in the affected segment. It is noted that a large number of patients treated surgically at HIAE presented with a previous indication of arthrodesis, and after evaluation, were submitted to simple decompression, impacting the cost of treatment.

The net cost of surgical treatment is confirmed as greater than the non-surgical, primarily when compared with the two first years of treatment. Nevertheless, patients in persistent conservative regime, especially with a diagnosis of degenerative spondylolisthesis and herniated disc, may show long-range costs similar or even greater than surgical patients^(16,^.

In this way, we note that in the treatment performed at a specialized center, precise surgical indication and constant reevaluation of the patient under conservative treatment are fundamental steps towards determining cost reduction in the treatment of spinal disorders.

## CONCLUSION

In literature, there is a small quantity of articles related to the costs of spinal surgery. More frequently evaluations of cost-effectiveness of the treatments may be found, but there are no references, on a large scale, as to the comparison of the costs of conservative *vs*. surgical treatment.

Analysis of these data allowed us to observe that, in caring for patients within a specialized center, there was a greater indication for conservative treatment and consequently, reduction in the final cost of treatment. It was observed that the subject needs to be better studied, since standardization of the indications of treatment would allow a better comparison of the costs between the different centers.
